# The Biotechnological Potential of Crickets as a Sustainable Protein Source for Fishmeal Replacement in Aquafeed

**DOI:** 10.3390/biotech13040051

**Published:** 2024-11-21

**Authors:** Aldo Fraijo-Valenzuela, Joe Luis Arias-Moscoso, Oscar Daniel García-Pérez, Libia Zulema Rodriguez-Anaya, Jose Reyes Gonzalez-Galaviz

**Affiliations:** 1 Programa de Doctorado en Ciencias Especialidad en Biotecnología, Instituto Tecnológico de Sonora, Ciudad Obregón 85000, Sonora, Mexico; aldo.fraijo231188@potros.itson.edu.mx; 2 Departamento de Ingeniería, Tecnológico Nacional de México, Instituto Tecnológico del Valle del Yaqui, Bácum 85276, Sonora, Mexico; jarias.moscoso@itvy.edu.mx; 3 Facultad de Medicina Veterinaria y Zootecnia, Universidad Autónoma de Nuevo León, Gral. Escobedo 66054, Nuevo León, Mexico; oscar.garciapr@uanl.edu.mx; 4 CONAHCYT-Instituto Tecnológico de Sonora, Ciudad Obregón 85000, Sonora, Mexico; libia.rodriguez@conahcyt.mx

**Keywords:** cricket meal, fishmeal, aquaculture, protein, growth, biotechnology

## Abstract

As aquaculture production grows, so does the demand for quality and cost-effective protein sources. The cost of fishmeal (FM) has increased over the years, leading to increased production costs for formulated aquafeed. Soybean meal (SBM) is commonly used as an FM replacer in aquafeed, but anti-nutritional factors could affect the growth, nutrition, and health of aquatic organisms. Cricket meal (CM) is an alternative source with a nutrient profile comparable to FM due to its high protein content, digestibility, and amino acid profile. CM use in aquafeed influences growth and reproductive performance while modulating the gut microbiota and immune response of fish and shrimp. However, consistent regulation and scaling up are necessary for competitive prices and the marketing of CM. Moreover, the chitin content in CM could be an issue in some fish species; however, different strategies based on food biotechnology can improve the protein quality for its safe use in aquafeed.

## 1. Introduction

Aquaculture is an alternative economic activity to produce marine food and resources due to the overexploitation of wild fish and shellfish [1]. In 2022, the annual global aquaculture production was 130.9 million tons, equal to USD 312.8 billion [2]. However, as a side effect of overfishing, aquaculture faces the challenge of finding sustainable protein sources for formulated aquafeed [2]. Fishmeal (FM) is the most important ingredient in formulated feed; it acts as the main protein source due to the essential amino acid content, which meets the protein requirements of most cultured species [2,3]. This endangers aquaculture’s sustainability since the cost of feed accounts for 50% of the total production costs [4]. Therefore, it is critical to find feasible alternatives to FM that meet the protein requirements of cultured species while being environmentally sustainable and cost effective [5,6]. Soybean meal (SBM), a common alternative source to FM, has a decent protein content, availability, and low cost; nevertheless, it contains anti-nutritional factors and lacks essential amino acids, disrupting feeding intake, causing gastrointestinal damage, impairing immune function and protein synthesis, reducing growth performance, and decreasing economic yields [7,8]. Meanwhile, cricket meal (CM) has a nutrient profile comparable to FM in terms of quality and quantity due to containing high-quality nutrients that are easily digestible and more bio-available than those found in SBM [9,10]. Therefore, this review article aimed to investigate the biotechnological potential of CM as a sustainable protein source for FM replacement in aquafeed, including its nutritional value and protein quality, effects on the growth and immunity of aquatic species, and challenges for its safe use as an ingredient in aquafeed.

## 2. The Nutritional Value of Cricket Meal

**Figure 1:**
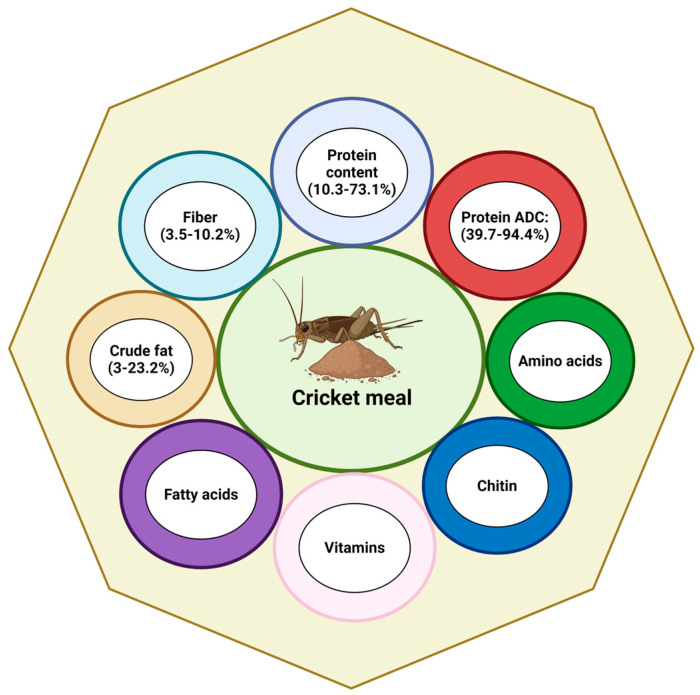
Nutritional composition of cricket meal. Created with BioRender.com (accessed on 9 October 2024).

Cricket (*Orthoptera*: *Gryllidae*) production is cost effective and profitable because crickets can be reared on various substrates, including organic waste, reducing costs and pollution. Moreover, they require less space and water than vegetable sources for SBM production [11,12,13,14,15,16,17]. For example, crickets require a small space (~15 m^2^) and 1.7 kg of feed to increase 1 kg of their body weight [15,17,18]. Moreover, crickets are highly efficient due to their rapid breeding cycles [19,20], offering the possibility to produce a renewable protein source that could meet the nutritional requirements of aquatic organisms. Therefore, crickets have emerged as a good protein source for FM replacement in aquafeed [21,22,23,24,25]. Previous studies indicate that CM contains substantial quantities of nutritionally valuable components, such as high crude protein rich in amino acids (AAs), good lipid sources, minerals, and vitamins [9]. Therefore, the CM from different cricket species (*Acheta domesticus*, *Gryllus assimilis*, and *Gryllus bimaculatus*) could be a sustainable alternative protein source for FM replacement in aquafeed [10] based on the nutritional requirements for major economically aquatic species [3,26]. However, before incorporating CM into aquafeed, its proximate composition must be analyzed and compared with the main protein sources in aquafeed [27]. A schematic diagram of the nutritional composition of crickets is illustrated in Figure 1. Additionally, the nutritional composition of CM compared with FM and SBM is presented in Table 1.

The protein content in aquafeed ranges from 25% to 60% crude protein (CP), and proteins constitute a significant portion of the body composition in fish and shrimp (65–85%) [26].

The CM protein content is usually very high and is comparable to that in FM and superior to that in SBM, which is one of the most common protein sources in aquafeed (Table 1). The protein quality should be determined according to the AA profile and apparent digestibility coefficients (ADCs) to assess alternative protein sources for aquafeed. Fish and shrimp require indispensable or essential amino acids (EAAs) and dispensable or non-essential amino acids (NEAAs) to sustain optimal growth performance, reproduction, health, and flesh quality [26,28]. FM is a valuable protein source in aquafeed due to its AA profile, but the limited supply and high cost of FM require research into alternative sources for aquafeed. SBM is the most common protein source for FM replacement but contains anti-nutritional factors and an EAA imbalance [29,30]. Regarding protein digestibility, FM has a protein ADC of 91.6% [31]; SBM has a protein ADC of 83% [32]; and CM has a protein ADC of up to 90.4% for fish and shrimp. In addition, CM has better EAA/NEAA ratio values than SBM. Therefore, CM could be a promising alternative for FM replacement due to its better AA profile and higher digestibility than SBM.

Lipids serve as a dense energy source, as structural components in membranes, and as carriers of essential fatty acids and are essential for fat-soluble vitamins [26,33]. Therefore, lipids supply the essential fatty acids (EFAs) required for fish and shrimp to develop different physiological functions, including reproduction, immunity, growth, and survival [34]. In aquatic organisms, the EFAs are mainly polyunsaturated fatty acids (PUFAs), such as α-linolenic acid (ALA) and linoleic acid (LNA), eicosapentaenoic acid (EPA), docosahexaenoic acid (DHA), and arachidonic acid (ARA), due to their limited biosynthetic ability [35]. Fish oil (FO) is the main lipid source in aquafeed due to its abundant n-3 polyunsaturated fatty acids, and it is crucial for the growth and reproduction of aquatic species [33]; however, FM contributes approximately 10% fat in addition to FO [35]. Due to fish raw material limitations, SBM utilization in aquafeed could decrease the ω3/ω6 ratio due to the high ω6 content and absence of EPA and DHA, compromising the fish’s health, nutritional quality, and sensory properties [36]. The lipid content in CM ranges from 3 to 46% crude fat, and CM has a lower ω6 content than SBM (Table 1). However, the source crickets for the CM could be fed ω3-enriched diets, or cricket-based aquafeed could be supplemented with ω3-enriched oil sources to increase the ω3/ω6 ratio. For example, dietary flaxseed oil increased the content of EPA in *A. domesticus* when fed at 1, 2, and 4% inclusion levels [37]. Studies on alternative lipid sources, such as fish oil replacement, are necessary, but these will not be covered in this review. Therefore, based on its proximate composition, CM should be a nutritionally valuable source with a high protein content, digestibility, and good EAA profile, making it suitable as an FM replacement in aquafeed.

**Table 1:** The proximate composition and protein quality of cricket meal made from different species, fishmeal, and soybean meal.

Composition (g/100 g, Dry Matter)	*A. domesticus*	*G. assimilis*	*G. bimaculatus*	FM	SBM
Protein	10.3–73.1	62.1–64.9	57.0–59.9	57.40–73	49.4–54.0
Lipids	3–22.8	18.14–23.2	13.9–46.0	4.7–9.9	0.9–1.8
Fiber	3.5–10.2	7.0–8.3	8.4–9.5	0.5	3.4–7.9
Carbohydrates	NA	8.6–12.5	NA	15.0	4.8–7.0
Ash	4.41–8.36	4.48–4.8	4.8–5.4	12.70–18	6.0–7.0
Moisture	4.72–73.2	NA	7.4	NA	10.4–12.5
EAA					
Arginine	3.73–8.53	4.04	4.59–6.20	3.7–7.42	3.59
Histidine	0.67–2.93	1.52	1.51–2.20	1.30–7.86	1.32
Isoleucine	2.0–5.31	2.91	1.88–3.75	2.60–5.04	2.17
Leucine	3.80–8.69	4.83	3.79–6.70	4.23–7.81	3.74
Lysine	3.22–6.16	3.90	3.02–5.14	4.49–8.78	3.16
Methionine	0.93–1.49	1.10	0.98 ± 2.02	1.51–2.93	0.82
Phenylalanine	1.36–4.23	2.34	1.75–4.0	2.21–5.38	2.50
Threonine	1.65–4.49	2.54	2.55–3.58	2.42–6.26	1.99
Tryptophan	0.38–0.68	0.68	0.25–0.85	0.62	0.72
Valine	2.76–6.99	3.84	2.85–4.24	2.88–5.56	2.27
NEEA					
Alanine	3.67–5.92	5.89	3.47	4.14	2.15
Aspartate	4.61–5.66	5.66	2.87	6.22	5.56
Cystine	0.40–1.17	0.55	2.02	0.65	0.98
Glutamate	6.2–7.26	6.48	6.77	8.36	9.32
Glycine	2.6–3.65	3.50	3.31	3.72	2.06
Proline	3.04–5.84	3.54	2.81	3.18	2.46
Serine	1.59–2.87	2.87	1.59	2.49	2.51
Tyrosine	2.71–4.91	3.18	7.63	2.20	1.87
EAA/NEAA	0.83–1.33	0.87	0.76–1.27	0.84–1.86	0.83
Protein ADC (%)	65.8–76.5	39.7	71.2–90.4	91.6	83.0
EFA					
ALA	41.39	26.13	4.15	0.54	6.8
LNA	NA	NA	NA	1.24	51.0
ARA	0.01	NA	0.01	0.84	0
EPA	0.01	NA	0	11.44	0
DHA	0.11	0.03	0.02	12.3	0
SFA	32.2	43.7	3.25	26.43	14.2
MUFA	21.7	27.5	3.13	35.26	30.0
PUFA	42.6	28.8	4.33	37.4	55.8
ω3	1.10	NA	1.13	27	6.8
ω6	32.91	NA	24.33	2.56	51.0
ω3/ω6	0.07	NA	0.04	10.8	0.13
References	[21,22,23,38,39,40,41,42,43,44]	[38,40,45]	[40,46,47,48,49,50]	[22,23,47,51]	[32,52]

NA: Not available.

## 3. The Dietary Use of Cricket Meal Influences the Growth Performance, Physiological Response, and Microbiota Composition of Different Aquatic Species

**Figure 2:**
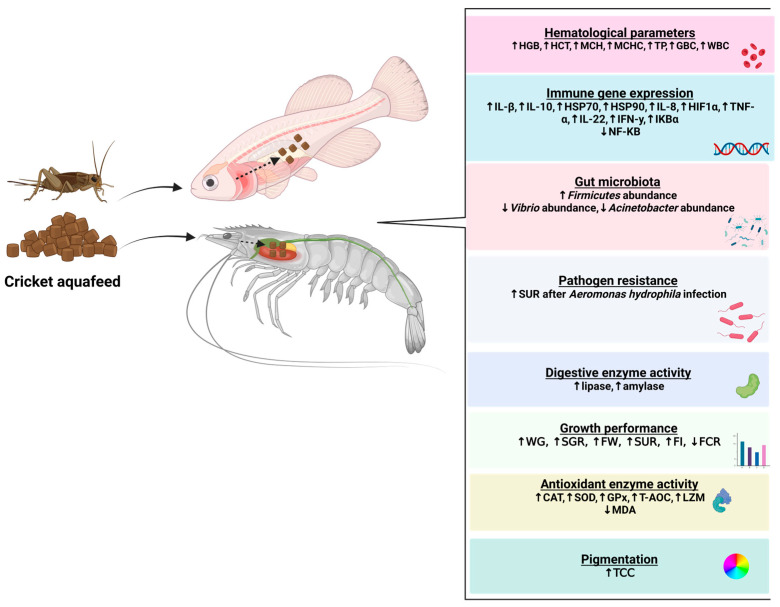
Illustration of cricket meal’s effects on aquaculture. Created with BioRender.com (accessed on 9 October 2024).

Crickets have a valuable nutritional profile and are considered a promising protein source for aquafeed formulations because they represent a sustainable and economically viable alternative for FM replacement. Table 2 summarizes a selection of studies examining the effects of CM as an FM replacer on some aquaculture species. An overall schematic representation of CM’s effects on aquaculture is shown in Figure 2.

CM inclusion in aquafeed has a positive effect on the growth performance, feed utilization, and other physiological responses of aquatic organisms. CM could replace FM in aquafeed, partially and completely, in different aquatic species: 10–50% in white shrimp (*Penaeus vannamei*), 20% in olive flounder (*Paralichtys olivaceus*), 25–80% in tilapia (*Oreochromis* spp.), 30% in Northern snakehead (*Chana argus*); 50–75% in channel fish (*Ictalurus punctatus*), up to 75% in guppy (*Poecilia reticulata*), and up to 100% in African catfish (*Claria gariepinus*), Indian carp (*Catla catla*), and striped snakehead (*Chana striata*). The differences in CM inclusion in aquafeed could be attributed to the nutrient requirements, nutrient utilization, physiology, and anatomical systems of aquatic organisms, specifically in chitin digestion contained in CM [48]. Therefore, FM replacement with CM depends on factors such as aquatic organism species, cricket species, life stage, and diet composition [22]. Another reason could be due to the presence of chitinolytic enzymes (chitinase, chitobiase, and lysozyme) in some aquatic organisms with carnivorous or omnivorous behavior [64]. Nevertheless, it is important to highlight that CM is an excellent protein source with a high nutritive value and protein quality in aquafeed.

Positive effects of CM inclusion in aquafeed have also been observed on the antioxidant activity, hematological parameters, immune-related genes, and microbial composition of aquatic organisms without negatively affecting the histology of the digestive organs. Antioxidant activity is a complex physiological response carried out by various enzymes such as superoxide dismutase (SOD), catalase (CAT), and glutathione peroxidase (GPx). These enzymes help organisms to prevent the accumulation of harmful reactive oxygen species (ROS) and protect cells from oxidative damage, while malondialdehyde (MDA) is an important indicator of oxidative stress [65]. Most studies in which FM was replaced with CM indicate that enzymes related to antioxidant activity increased and MDA decreased, suggesting improved health and reduced oxidative stress in aquatic organisms. Hematological parameters are reliable indicators of fish physiology and health under different nutritional or environmental conditions, as increased hemoglobin (HGB) levels enhance oxygen delivery to tissues, and higher HGB and hematocrit (HCT) levels help eliminate carbon dioxide from the body [62]. The studies in which CM was used as a protein source for FM replacement indicated that the oxygen-carrying capacity of the aquatic organisms’ blood was improved, which could influence their growth performance. Interleukins and heat shock proteins are important in maintaining tissue and immune homeostasis. Interleukins have pro- and anti-inflammatory functions, and heat shock proteins have stressor protection [60]. The observed data in this review suggest that using CM as a replacement for FM in aquafeed leads to an upregulation of immune-related genes, thereby improving the immune response of aquatic organisms. The upregulation of immune-related genes may be due to gene expression plasticity in the short- and medium-term as a nutritional adaptation response in long-term nutritional evaluations [66]. Additionally, the aquatic organisms’ microbiota has key functions in the digestion and absorption of nutrients, immune response, and protection against harmful invaders [67]. That review noted that CM inclusion increases beneficial bacteria and reduces pathogenic bacteria in the microbiota composition of Northern snakehead and catfish. However, this effect might be due to the largely unknown influence of CM on the gut microbial community of aquatic animals. Therefore, more research is necessary to investigate the optimal CM level in aquafeed and its effects on the growth performance and welfare of aquatic organisms.

## 4. Challenges, Limitations, and Possible Solutions

### 4.1. Environmental Impact and Economic Feasibility of Cricket Meal Production

CM production is relatively fast and has a minimal environmental impact compared with FM and SBM production (Table 3).

FM production is energy intensive and contributes to water pollution due to its phosphorus content [68]. Climate phenomena, such as the El Niño, influence the fish availability for FM production [73], which increases costs. Plant-based protein sources are commonly used as alternative protein sources in aquaculture [74]; however, their production causes land use intensification, increased freshwater consumption, and higher greenhouse emissions [75], which also have climate change consequences [76]. Plant-based protein sources release more phytate phosphorus and increase nitrogen excretion in the aquatic environment [77], making them unfit FM replacers in aquafeed. Meanwhile, CM demonstrates reduced nitrogen and phosphorus excretion in aquatic environments [48]. Additionally, life cycle analysis (LCA) demonstrates the environmental efficiency of cricket production [78,79], showing an efficient balance between land and water use with a moderate global warming potential, making CM a viable choice for environmentally friendly aquafeed despite its high energy use. Regarding economic feasibility, FM and SBM production volumes are much higher than those of CM production, with market prices of USD 1.91/Kg, USD 0.32/Kg, and USD 40/Kg, respectively [80,81]. In addition, legislation and regulations limit global cricket production and marketing due to regulation inconsistencies across international borders and limited biowaste substrate options for cricket rearing [82,83]. Therefore, it is necessary to define consistent regulations with safe sanitation procedures to scale up CM production and compete with the prices of commonly used protein sources in aquafeed formulations [23,84].

### 4.2. Challenges in Cricket-Meal-Based Aquafeed

**Figure 3:**
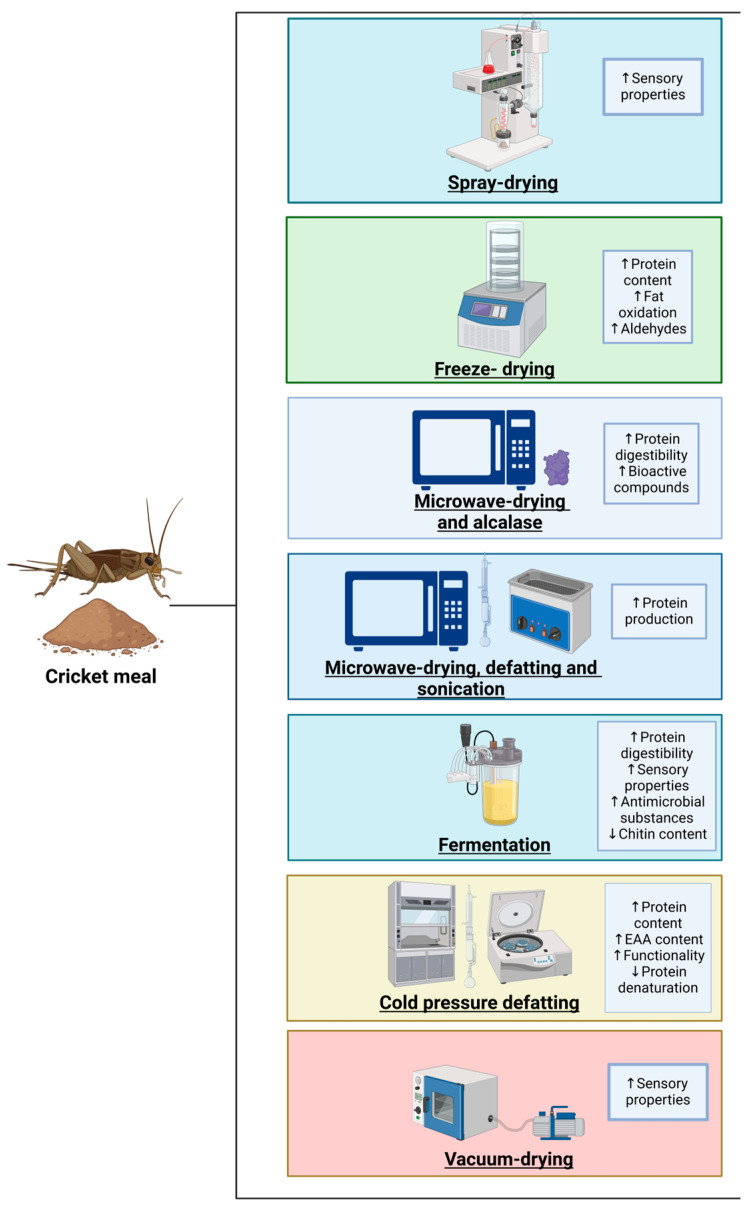
Illustration of different processing methods to improve protein quality of cricket meal. Created with BioRender.com (accessed on 9 October 2024).

Chitin is the main component of the cricket exoskeleton. Elevated chitin levels decrease the protein digestibility and functional properties of CM as an aquafeed ingredient [64,85]. Moreover, the most common method to obtain CM involves oven-drying whole crickets, followed by grinding them with an industrial food processor, resulting in particles larger than 300 µm due to the presence of chitin. This issue reduces palatability, negatively affecting sensory acceptance in feeds [86]. An alternative to decrease the chitin presence is to produce a finer CM via wet blending, followed by spray drying, which improves sensory characteristics [87]. Moreover, it has been reported that freeze drying increases the protein content in insect meal but also increases aldehydes and fat oxidation, affecting the CM’s palatability; microwave drying produces moderate protein quality; and vacuum drying reduces the protein quality [88]. Nevertheless, microwave radiation combined with alcalase activity enhances the bioactive peptides, consequently increasing the protein digestibility of CM [89]. Regarding enzymatic hydrolysis, *Yarrowia lipolytica* and *Debaryomyces hansenii* have been used to produce CM hydrolysates with a lower chitin content, higher antibacterial substances, health-promoting molecules, enhanced protein digestibility, and improved sensory properties [90]. Another study reported that microwave drying crickets, followed by hexane defatting combined with sonication, improved the protein production from CM, but the protein quality was not determined [91]. In addition, defatting methods for protein production from insect meal have shown differences in protein quality since cold pressure produces higher protein contents with increased amounts of EAAs, better functionality, and low protein denaturation compared with solvent extraction [92]. Nevertheless, chitin in lower quantities could act as a prebiotic in aquatic organisms, suggesting that chitin inclusion in aquafeed could increase beneficial bacterial diversity in the gut, which stimulates gut fermentation and produces essential short-chain fatty acids that support the growth performance, intestinal health, immune response, resistance against pathogens, and welfare of aquatic organisms [93,94]. Therefore, the low chitin content in CM could be used advantageously in aquafeed supplemented with chitinase or chitinolytic probiotics [64] to improve the growth performance and health of aquatic organisms. Nonetheless, more food biotechnology research is necessary to improve the protein quality and sensory properties of CM for safe use in aquafeed. Different processing methods applied to improve CM’s protein quality are shown in Figure 3.

## 5. Conclusions

Cricket meal emerges as a feasible, cost-effective, and sustainable protein source alternative to fishmeal due to its high protein content, digestibility, and amino acid profile. Therefore, cricket meal could serve as a valuable ingredient in aquafeed. Cricket meal inclusion could improve the growth and welfare of aquaculture species, but more investigation is required to determine the optimal inclusion level in aquafeed. Cricket farming requires consistent regulations to scale up cricket meal production and reach competitive marketing prices. Food biotechnology research is necessary to improve the protein quality of cricket meal for its safe use in aquafeed.

## Figures and Tables

**Table 2:** Cricket meal’s effects on growth performance, antioxidant capacity, and immune gene expression.

Aquatic Species	Initial Weight (g)	Bioassay Duration (Days)	Cricket Species	Optimal CM Inclusion (%)	CM Inclusions (%)	Effects	References
*Penaeus vannamei*	0.17	65	*Gryllus bimaculatus*	10	10% CM CD: 250 g/kg FM.	Performance: No differences in FW, SGR, FCR, and SUR between CM and FM basal diet; ↑FI. Antioxidant enzyme activities: no differences in PO, SOD, and GPx; ↑NBT activity in 10% CM.	[53]
*Penaeus vannamei*	0.73	40	*Gryllus bimaculatus*	50	0%, 10%, 20%, 30%, 40%, and 50% CM. CD: 426.10 g/kg FM.	Performance: ↑WG, ↑SGR, ↑SUR, and ↓FCR in 50% CM.	[54]
*Clarias gariepinus*	4	56	*Gryllus bimaculatus*	100	0%, 25%, 50%, 75%, and 100% CM. CD: 300 g/kg FM.	Performance: ↑FW in 50, 75, and 100% CM with highest value in 100% CM; ↑WG in 75 and 100% CM with highest value in 100% CM; ↑SGR in 100% CM; ↓FCR in 100% CM; no differences in SUR between groups.	[50]
*Clarias gariepinus*	13.2	49	*Gryllus bimaculatus*	100	0%, 75%, and 100% CM. CD: 350 g/kg FM.	Performance: ↑FI, ↑WG, ↑SGR, and ↑SUR in 100% CM with lowest FCR in 100% CM. Antioxidant enzyme activities: ↑CAT in 100% CM; no differences in GST and SOD between groups.	[55]
*Chana striata*	15	70	*Gryllus bimaculatus*	100	100% CM. CD: 450 g/kg FM.	Performance: ↑FW and ↑WG in 100% CM; ↓FCR in 100% CM; no differences in FI, SGR, and SUR between the FM diet and CM diet.	[49]
*Chana striata*	15	70	*Gryllus bimaculatus*	100	0%, 50%, and 100% CM. CD: 450 g/kg FM.	Performance: ↑FW, ↑WG, and ↓FCR in 100% CM; no differences in SGR between dietary groups.	[48]
*Paralichtys* *olivaceus*	33.5	56	*Gryllus bimaculatus*	20	0, 20, 40, 60, and 80% CM. CD: 650 g/kg FM.	Performance: ↑WG in 20% CM; lowest SGR in 80% CM; no differences in SUR and FCR between dietary groups. Antioxidant enzyme activities: ↑SOD and ↑GPx in 60% CM; no differences in LZM and MPO activities between dietary groups.	[56]
*Oreochromis niloticus*	8	21 (sex reversal treatment)	*Gryllus bimaculatus*	60–80	0, 20, 40, 60, 80, and 100% CM. CD: 987 g/kg FM.	Performance: ↑SUR; ↓FCR in 80% CM; no differences in SGR and FW between groups.	[57]
*Oreochromis niloticus*	0.30	30 (nursery I)	*Gryllus bimaculatus*	60–80	0, 20, 40, 60, 80, and 100% CM. CD: 382 g/kg FM.	Performance: ↑FW in 60% and ↓FCR in 60% CM; ↑SUR in 80% CM; ↑SGR in 40% CM.	[57]
*Oreochromis niloticus*	0.33	33 (nursery II)	*Gryllus bimaculatus*	60	0, 20, 40, 60, 80, and 100. CD: 382 g/kg FM.	Performance: ↑FW and ↓FCR in 60% CM; no differences in SGR between dietary groups.	[57]
*Oreochromis* spp.	7.9	98	*Gryllus bimaculatus*	25–50	0, 25, 50, and 75% CM. CD: 200 g/kg FM.	Performance: No differences in FW, WG, and SGR between 25 and 50% CM in the FM diet (0%); no differences in SUR and FCR between dietary groups.	[47]
*Poecilia reticulata*	0.0057	30	*Gryllus bimaculatus*	75	0, 50, 75, and 100% CM. CD: 450 g/kg FM.	Performance: No differences in FW, WG, SGR, and SUR between dietary groups. Pigmentation: ↑TCC in 100% CM.	[42]
*Poecilia reticulata*	0.0057	30	*Acheta domesticus*	75	0, 50, 75, and 100% CM. CD: 450 g/kg FM.	Performance: No differences in FW, WG, SGR, and SUR between dietary groups. Pigmentation: No differences in TCC between dietary groups.	[42]
*Oreochromis* sp.	5	60	*Gryllus assimilis*	32	68% CM. CD: 32% CP in commercial diet.	Performance: No differences in SUR, FCR, and FB between dietary groups.	[58]
*Oreochromis* sp.	1.427	28	*Acheta domesticus*	60	0, 60, 70, 80, 90, and 100% CM. CD: commercial diet (Cargill, Malaysia).	Performance: ↑SGR in 60% CM; no differences in FW between groups.	[59]
*Chana argus*	3.5	70	*Gryllus testaceus*	30	0, 15, 30, 45, and 60% CM. CD: 450 g/kg FM.	Performance: No differences in FW, WG, FCR, and SGR in 15% and 30% CM compared with the control diet (0% CM); no differences in SUR and FI between groups. Antioxidant enzyme activities: No differences in CAT, GPx, and SOD between groups; ↑T-AOC in 30% and 45% CM with highest value in 45%; lowest MDA in 45 and 60% CM with lowest value in 60% CM. Immune gene expression: ↑IL-β, ↑IL-10, ↑HSP70 ↑HSP90, and ↓IL-8 in all CM groups; ↑IκBα in 30 and 45% CM. Histology: No differences in mid-intestinal thickness in 15, 30, and 45% CM compared with the control (0% CM); ↓villus height in CM groups; no differences in villus width between groups. Digestive enzymes activities: ↑Amylase activity in 60% CM; ↑lipase activity in 45 and 60% CM with highest value in 45% CM. Gut microbiota: ↑*Firmicutes* abundance in 60% CM.	[60]
*Catla catla*	0.22	*56*	*Gryllus bimaculatus*	100	0, 35, 70, and 100% CM. CD: 250 g/kg FM.	Performance: ↑FW and ↑FI in 70 and 100% CM with highest value in 100% CM; no differences in WG, SUR, and FCR between dietary groups. Liver and gill histology: No changes were observed in gill and liver tissues between groups. Bacterial challenge: No differences in SUR between groups.	[61]
*Ictalurus punctatus*	2.7	70	*Gryllus bimaculatus*	50–75	0, 25, 50, 75, and 100% CM. CD: 350 g/kg FM.	Performance: ↑FW, ↑WG, and ↑SGR in 50 and 75% CM with highest values in 75% CM; no differences in FI and SUR between treatments. Hematological parameters: No differences in WBC, RBC, MVC, PLT, and MPV between groups; ↑HGB, ↑HCT, and ↑MCH in all CM groups with highest values in 50% CM; ↑MCHC in all CM groups with highest values in 100% CM. Antioxidant enzyme activities: No differences in SOD and T-AOC between groups; no differences in MDA between 0% CM and 100% CM; ↑MDA in 75% CM; ↑CAT in 50, 75, and 100% CM with highest values in 50% CM; ↑GPx in 75% CM. Immune gene expression: ↑IL-1β, ↑IL-8, and ↑IL-10 in 25, 50, and 100 CM groups with highest values in 75%; ↑TNF-α in 100% CM; ↑IL-22 and ↑IFN-y in 50, 75, and 100% CM groups with highest values in 100% CM; ↓NF-KB in 50, 75, and 100% CM groups with lowest value in 100% CM; ↑HIF1α in 75% CM. Gut microbiota: ↑*Firmicutes* abundance in CM groups; ↓*Vibrio* abundance in 100% CM; ↓*Acinetobacter* abundance in 50 and 100% CM.	[62]
*Oreochromis niloticus*	9	40	*Acheta domesticus*	30	20 and 35% CM. CD: Commercial diet (Nutripec, Cargill)	Performance: No difference in FW, FB, and MR between the control and 35% CM.	[12]
*Clarias gariepinus*	22.5	40	*Gryllus bimaculatus*	40	35 and 40% CM. CD: 350 g/kg FM	Hematological parameters: ↑TP and ↑GBC in 35% and 40% CM with highest values in 40% CM; ↑WBC in 40% CM. Antioxidant enzyme activities: ↑LZM in 35 and 40% CM with highest values in 40% CM. Bacterial challenge: ↑SUR in both CM groups with highest value in 40% CM; ↓MR in both CM groups with lowest value in 40% CM.	[63]

CM: cricket meal; FM: fishmeal; CD: control diet; FW: final weight; WG: weight gain; SGR: specific growth rate; FI: feed intake; FCR: feed conversion ratio; SUR: survival rate; FB: final biomass; MR: mortality rate; WBC: white blood cell count; TP: total protein; GBC: globulin concentration; LZM: lysozyme activity; SOD: superoxidase dismutase; GST: glutathione S-transferase; CAT: catalase; MPO: myeloperoxidase; TLG: total immunoglobin; GPx: glutathione peroxidase; NBT: nitro-blue tetrazolium; PO: phenoloxidase; T-AOAC: total antioxidant capacity; TCC: total carotenoid concentration; RBC: red blood cell; MVC: mean corpuscular volume; MCHC: mean corpuscular hemoglobin concentration; PLT: platelet; MPV: mean platelet volume; HGB: hemoglobin; HCT: hematocrit; MCH: mean corpuscular hemoglobin; MCH: mean corpuscular hemoglobin; MDA: malondialdehyde; IL-1β: interleukin-1β; IL-8: interleukin-8; IL-10: interleukin-10; IL-22: interleukin-22; TNF-α: tumor necrosis factor-α; IFN-γ: interferon-γ; HSP70: heat shock protein 70; HSP90: heat shock protein 90; NF-κB p65: nuclear factor kappa-B p65; IκBα: nuclear factor kappa B inhibitor protein; IFN-y: interferon gamma; ↑: significantly increased; ↓: significantly decreased.

**Table 3:** Environmental impact of cricket production compared with fishmeal and soybean production.

Protein Source	Energy Use (MJ/Kg)	Land Use (m^2^/Kg)	Water Use (L/g protein)	Global Warming Potential (Kg CO_2_eq/Kg protein)	References
Fishmeal	10.52	0.00055	0.087	0.265–0.576	[68,69]
Soybean	4.1	8.7	19.1	5.2	[70]
*A. domesticus*	21.1	0.12	2	4.2	[70,71,72]

## Data Availability

No new data were created or analyzed in this study. Data sharing is not applicable to this article.
